# Multiple sclerosis and COVID-19: a bidirectional Mendelian randomization study

**DOI:** 10.3389/fimmu.2024.1451347

**Published:** 2024-10-18

**Authors:** Shitong Liu, Yixin Liang, Binbin Sheng, Rongxin Zhang

**Affiliations:** ^1^ School of Basic Medical Sciences, Guangdong Pharmaceutical University, Guangzhou, China; ^2^ School of Life Sciences and Biopharmaceutics, Guangdong Pharmaceutical University, Guangzhou, China; ^3^ Guangdong Provincial Key Laboratory of Advanced Drug Delivery, Guangdong Provincial Engineering Center of Topical Precise Drug Delivery System, Guangdong Pharmaceutical University, Guangzhou, China

**Keywords:** COVID-19, multiple sclerosis, Mendelian randomization, single nucleotide polymorphism, interferon beta

## Abstract

This study aimed to investigate the potential relationship between multiple sclerosis (MS) and coronavirus disease 2019 (COVID-19) outcomes using Mendelian randomization analysis. Specifically, it evaluates whether genetic factors, including the single-nucleotide polymorphism (SNP) rs10191329, influence the susceptibility of MS patients to three COVID-19 outcomes [severe acute respiratory syndrome coronavirus 2 (SARS-CoV-2) infection, hospitalized COVID-19, and severe COVID-19]. This study utilized genome-wide association study summary statistics from the International Multiple Sclerosis Genetics Consortium to conduct a Mendelian randomization analysis. SNPs strongly associated with MS were selected to examine their impact on COVID-19 outcomes. The analysis focused on identifying any causal associations between MS and COVID-19 severity, as well as assessing the role of interferon beta (IFNβ) treatment in modifying these outcomes. The results suggest a potential association between MS and an increased risk of COVID-19, but individuals carrying the rs10191329 SNP appeared less likely to develop severe COVID-19. This SNP, located within the DYSF-ZNF638 locus, may influence immune responses and MS severity, highlighting its relevance for personalized treatment strategies. Importantly, no significant causal relationship was found between IFNβ treatment and the three COVID-19 outcomes, indicating that the findings in treated patients differ from those observed in untreated patients. This suggests that IFNβ may offer protective effects against SARS-CoV-2 in MS patients. These findings underscore the importance of genetic factors, such as rs10191329, in shaping the clinical outcomes of MS patients in the context of COVID-19. Further research should explore targeted therapies and personalized approaches for managing MS during the ongoing pandemic.

## 1 Introduction

In 2019, the coronavirus disease 2019 (COVID-19), caused by severe acute respiratory syndrome coronavirus 2 (SARS-CoV-2), spread rapidly around the globe, posing an unprecedented threat to global health (1, 2). Infection with SARS-CoV-2 can result in severe respiratory illnesses and may lead to serious complications in affected individuals (3). As of May 19, 2024, regarding the number of COVID-19 cases reported to the WHO (https://data.who.int/dashboards/covid19/cases?n=c), SARS-CoV-2 has resulted in a cumulative total of 775,522,404 confirmed cases and 7,049,617 deaths, raising health concerns for people with multiple sclerosis (MS).

MS is an autoimmune disease of the central nervous system. It is characterized by the immune system mistakenly attacking the myelin sheath, which protects the nerves. This results in impaired nerve function. Experimental studies have demonstrated that coronaviruses may cause demyelination in animal models and, at least in some cases, cause relapses of MS, possibly through a mechanism of molecular mimicry (MM) that triggers or exacerbates autoimmune diseases in the central nervous system (CNS) (4–6). In the treatment of MS, interferon beta (IFNβ) is a commonly used disease-modifying therapy (DMT) that reduces the incidence of attacks and inflammation on neural tissue by modulating the immune system. In particular, in patients with relapsing MS, it can slow disease progression and reduce relapses by modulating the immune system (7).

Mendelian randomization (MR) is a widely utilized methodology in the investigation of neurological disorders, explored to elucidate the causal relationship between exposure and outcome (8). The use of single-nucleotide polymorphisms (SNPs) as instrumental variables allows MR to follow the established statistical paradigms and to overcome the major challenges of assessing causality in observational studies, including reverse causality and confounding (2). MR has been extensively employed in the investigation of neurological disorders. The relationship between MS and COVID-19 has received considerable attention. There is an urgent need to clarify the relationship between COVID-19 and MS. In this study, we employed a two-sample MR approach using publicly available genome-wide association study (GWAS) data to investigate the potential causal relationship between MS and COVID-19, as well as between MS treated by IFNβ and COVID-19.

In the context of the increasingly prominent COVID-19 epidemic, no MR analysis has yet elucidated the association between MS patients receiving different treatments and COVID-19 (9), which is complemented by our further longitudinal study. In the horizontal study on the potential causal relationship between MS and COVID-19, the study of Baranova et al. showed that genetic predisposition to hospitalized COVID-19 had a causal effect on MS (2), which is consistent with the findings of this study. Although a study by Li et al. did not find a causal effect of severe COVID-19 on MS, their findings suggest that MS may be one of the risk factors for severe COVID-19 (9). This study shows that MS patients with rs10191329 have a lower risk of contracting severe COVID-19. Patients with the rs10191329 allele may experience less severe disease manifestations if they contract COVID-19.

## 2 Methods

### 2.1 Data sources

The GWAS summary statistics of the study samples undergoing IFNβ treatment consist of two independent cohorts from Sweden and Germany, with patients primarily from Northern and Central Europe, providing a certain level of geographical representation (https://gwas.mrcieu.ac.uk/) (**Supplementary Table 1**) (7). The summary statistics of COVID-19 outcomes were obtained from the COVID-19 Host Genetics Initiative (HGI) GWAS meta-analysis round 7 (publication date April 8, 2022, WITHOUT the 23andMe cohort), including SARS-CoV-2 infections (122,616 cases and 2,475,240 controls), hospitalized COVID-19 (32,519 cases and 2,062,805 controls), and severe COVID-19 (13,769 cases and 10,724,442 controls). The MS-GWAS dataset [GWAS data for MS provided by the International Multiple Sclerosis Genetics Consortium (IMSGC)] included 12,584 MS patients who participated in the GWAS (10, 11). In order to replicate the associations of the findings, the investigators replicated their findings in an additional 9,805 MS patients (11). Ethical approval was obtained for all original studies.

### 2.2 Data preparation and processing

Regarding data preparation and processing, the MR analysis was conducted using the “TwoSampleMR” package version 0.6.8. All analyses were based on the Genome Reference Consortium Human Build 37. The present study began by reading and processing data from the COVID-19 HGI exposure data, including SNP identifiers, effect value (beta), standard error, effect allele, other allele, p-value, sample size, and effect allele frequency. To reduce the linkage disequilibrium (LD) of SNPs, SNPs were clustered using PLINK software version 1.90b6.10, with a clumping distance set at 10,000 kb, an r² threshold at 0.001, and a p-value threshold at 5e−8.

### 2.3 Mendelian randomization analysis

In MR analysis methods, inverse variance weighted (IVW) or Wald ratio was chosen as the main method for MR analysis based on the number of SNPs, while additional sensitivity analyses were performed using methods such as MR-Egger, weighted median, and weighted models to ensure the robustness of the results (10). To ensure the robustness of the results, several sensitivity analyses were performed. First, pleiotropy was assessed using the intercept of the MR-Egger regression, which evaluates whether horizontal pleiotropy could bias the MR results. Cochran’s Q test was applied within the IVW method to examine heterogeneity among the genetic variants. Additionally, the MR-PRESSO method was employed for outlier detection and correction, with SNPs showing p-values less than 0.05 in the MR-PRESSO Outlier Test being excluded (9).

Heterogeneity was further assessed to determine whether significant variability existed in the SNP effects, thereby ensuring the credibility of the analysis. A leave-one-out analysis was conducted to evaluate the influence of individual SNPs on the overall results. By systematically removing each SNP and recalculating the MR estimates, we assessed whether any single SNP significantly altered the findings, thus testing the robustness of the results (10).

The final MR results were expressed as an odds ratio (OR) and presented in a variety of graphical presentations, including scatter plots, forest plots, sensitivity analysis plots, and funnel plots, to visualize the results of the analysis and its robustness. Using the above methods, the reliability and robustness of the MR analysis results were ensured, which provided solid evidence to investigate the potential causal relationship between COVID-19 and MS.

## 3 Results

**Figure 1:**
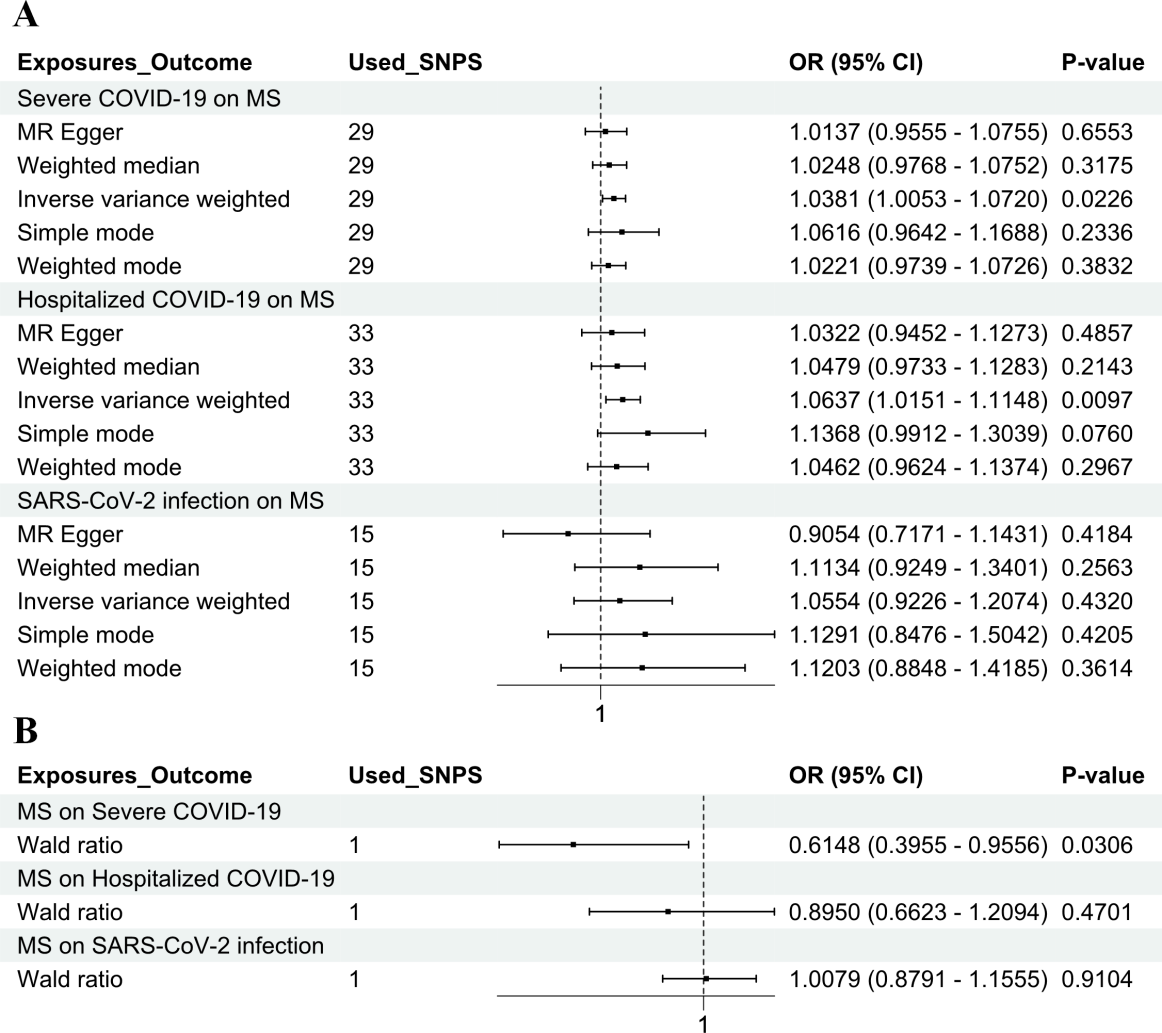
Genetic causality between COVID-19 and MS in a European population. **(A)** MR estimation of the effect of three COVID-19 exposure factors (SARS-CoV-2 infection, hospitalized COVID-19, and severe COVID-19) on MS as an outcome. **(B)** MR estimation of the effect of MS as an exposure factor on three COVID-19 outcomes (SARS-CoV-2 infection, hospitalized COVID-19, and severe COVID-19). The inverse variance weighted method and the Wald ratio method were considered as the main methods. COVID-19, coronavirus disease 2019; MS, multiple sclerosis; MR, Mendelian randomization; SARS-CoV-2, severe acute respiratory syndrome coronavirus 2.

In the forward MR analysis depicted in **Figure 1**, the three COVID-19 phenotypes were considered as the exposure, and MS was considered as the outcome. The IVW method was employed to ascertain the potential causal effect of hospitalized COVID-19 and severe COVID-19 on MS (IVW method: OR = 1.0637, 95% CI = 1.0151–1.1148, p = 0.0097; OR = 1.0381, 95% CI = 1.0053–1.0720, p = 0.0226). However, the IVW method did not find a significant effect of SARS-CoV-2 infection on MS (IVW method: OR = 1.0554, 95% CI = 0.9226–1.2074, p = 0.4320) (**Supplementary Tables 2**–**5**).

The MR analysis was performed using the “TwoSampleMR” package, with SNPs clumped via PLINK to reduce LD. A stringent threshold (r² < 0.001, 10,000 kb clumping distance, p < 5e−8) was applied to select SNPs strongly associated with MS. In the reverse MR analysis, MS was considered as the exposure, and the three COVID-19 phenotypes were considered as the outcomes. After filtering, rs10191329 was the only genome-wide significant SNP for MS, making it the most suitable instrument for the reverse analysis. This SNP, previously linked to MS in GWAS, was analyzed for its causal effect on COVID-19 severity using the Wald ratio method, revealing a potential association. The Wald ratio method is a straightforward MR approach used when only one genetic variant (SNP) is available as an instrument. In this case, rs10191329 was the only SNP identified that met the selection criteria for the reverse MR analysis. A potential causal association between MS and severe COVID-19 was identified (Wald ratio method: OR = 0.6148, 95% CI = 0.3955–0.9556, p = 0.0306) but has no significant causal association with SARS-CoV-2 infection and hospitalized COVID-19 (Wald ratio method: OR = 1.0079, 95% CI = 0.8791–1.1555, p = 0.9104; OR = 0.8950, 95% CI = 0.6623–1.2094, p = 0.4701) (**Supplementary Tables 2**–**5**).

In the bidirectional MR analysis shown in **Supplementary Figure 1** and **Supplementary Figure 2**, the results show that there was no significant causal relationship between any of the three COVID-19 exposures (SARS-CoV-2 infection, hospitalized COVID-19, and severe COVID-19), and MS patients were treated with IFNβ (**Supplementary Tables 6**-**8**).

In summary, the results of our MR study indicate that MS may reduce the severity of severe COVID-19 and that hospitalized COVID-19 and severe COVID-19 may increase the risk of MS. Nevertheless, the three types of COVID-19 were not causally related to MS patients treated with IFNβ.

## 4 Discussion

IFNβ, as a class of biopharmaceuticals used to treat MS, has been shown to act in a number of ways, including reducing the number of MS relapses, slowing disability progression, and potentially influencing the long-term course of the disease (7). It has been suggested that in the absence of treatment, patients with risk alleles may have disease progression at a rate similar to those treated with beta interferon (11). The time to reach a score of 6.0 on the Expanded Disability Status Scale (EDSS), which indicates the need for a walking aid (such as a cane) to walk 100 m, was shortened by 3.7 years in carriers of the rs10191329 risk allele compared to non-carriers, indicating a significantly faster disease progression. In contrast, patients receiving IFNβ treatment experienced a 4-year delay in reaching EDSS 6.0 (the median time in the treated group was 12.5 years, compared to 8.4 years in the untreated group), demonstrating that the treatment significantly slowed disease progression (12). Although the two groups showed opposing directions in disease progression, they exhibited similar rates of change.

**Figure 2:**
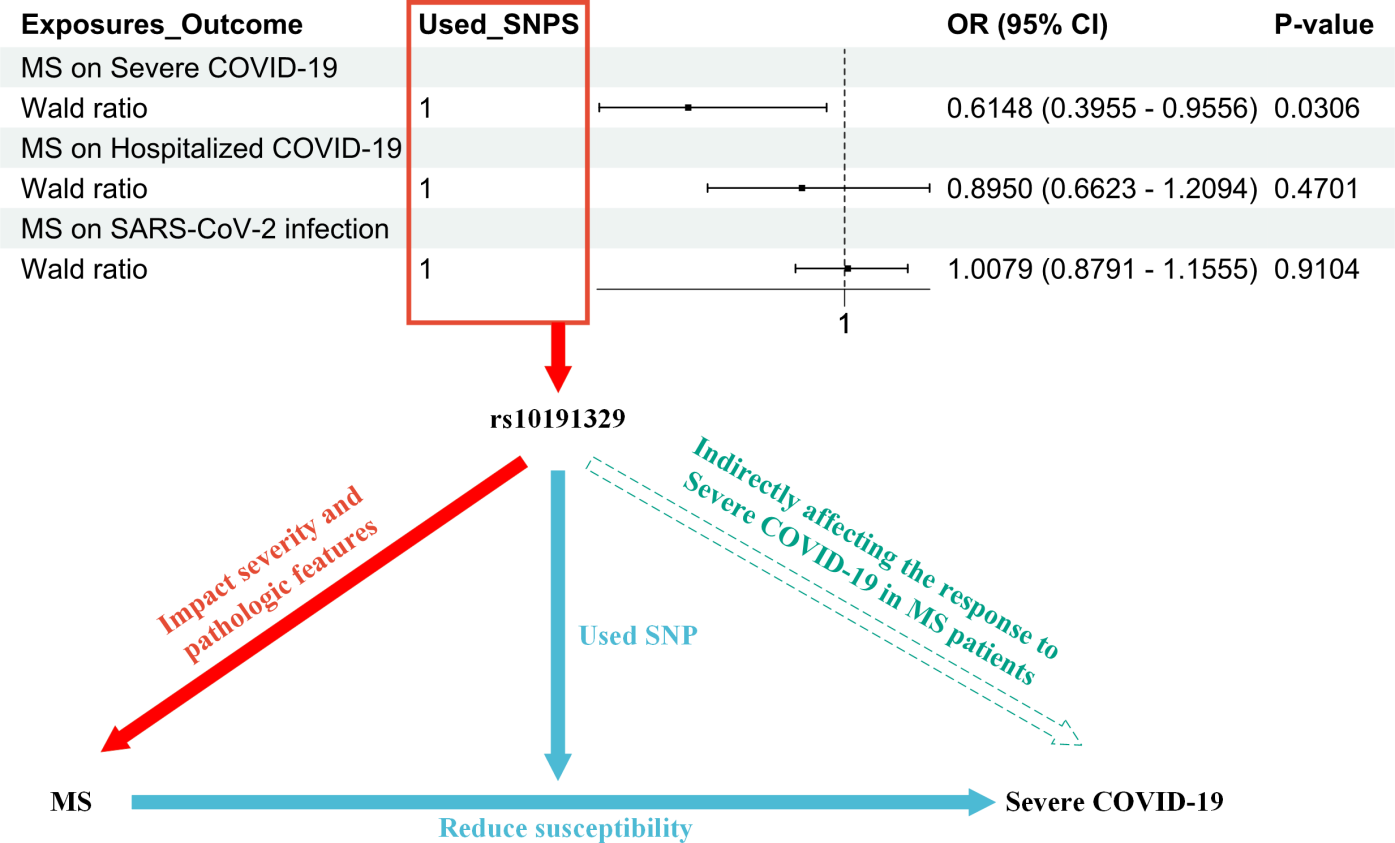
The figure illustrates the research process that led to the hypothesis that rs10191329 acts as a mediator; i.e., rs10191329 may indirectly influence the response of MS patients to COVID-19 by influencing the severity of MS and pathological characteristics. In the red section, it indicates that rs10191329 was involved as an instrumental variable in the MR analysis of MS as an exposure factor. This SNP has been shown to be associated with disease severity and pathological features of MS, which include faster disease progression and axonal loss as well as neuronal loss, which are key factors driving MS disease progression. The blue section demonstrates how MS disease severity increased susceptibility to severe COVID-19 when rs10191329 was used as an instrumental variable. This section emphasizes that MS severity may influence a patient’s response to severe COVID-19 infection. In the green section, a hypothesis is proposed that rs10191329 may indirectly influence the response of MS patients to severe COVID-19 by influencing MS severity and pathological characteristics. This hypothesis is based on the known association of rs10191329 with MS severity and takes into account that MS pathological features may influence patients’ immune status and reduce susceptibility to COVID-19. MS, multiple sclerosis; COVID-19, coronavirus disease 2019; SNP, single-nucleotide polymorphism.

In summary, IFNβ therapy is designed to slow the progression of MS and reduce recurrence and is effective for many patients. It is possible that patients who carry the rs10191329 (SNP) risk allele may experience faster disease progression even with IFNβ therapy. This suggests that genetic factors play an important role in disease severity and progression (11). It is worth noting that our study indicates that rs10191329 may indirectly influence the response of MS patients to COVID-19 by affecting the severity and pathological features of MS (**Figure 2**).

In this study, first, a bidirectional two-sample MR analysis was performed to explore the causal relationship between MS and COVID-19. The forward analysis identified a potential causal effect of hospitalized COVID-19 and severe COVID-19 on MS, suggesting that COVID-19 may be a risk factor for the development of MS. That is, infection with COVID-19, especially severe infection, may increase the risk of developing MS. The reverse analysis indicated a potential causal effect of MS on severe COVID-19, implying that MS patients with rs10191329 may be less susceptible to severe COVID-19 infections (**Figure 1**). Second, a bidirectional two-sample MR analysis was conducted using pooled statistics of participating MS from GWAS to investigate the potential causal relationship between MS patients treated with IFNβ and three types of COVID-19 phenotypes (SARS-CoV-2 infection, hospitalized COVID-19, and severe COVID-19). This suggests that the causal relationship between treated MS and COVID-19 has been reversed and that the treatment process may mitigate the negative impact of COVID-19 on MS. This finding requires further validation through additional experimental studies; for example, the research of Bellucci et al. demonstrated that IFNβ exerted protection against SARS-CoV-2 in people with MS (13) (**Supplementary Figures 1**, **2**).

The study demonstrated that rs10191329 was associated with the severity of MS. Specifically, the genetic variant in rs10191329 was found to be significantly associated with MS severity. MS patients carrying this risk allele may progress more rapidly to a stage where walking aids are required (11). Furthermore, rs10191329 has been linked to the pathological features of MS. Individuals who carry the purebred risk allele carriers exhibit more pronounced MS-specific brainstem and cortical pathological features, including axonal loss and neuronal loss, which are key factors driving MS disease progression (11) (**Figure 2**).

In addition, the results of the MR analysis of MS as an exposure factor and the outcomes of COVID-19 demonstrated a potential causal relationship between MS and severe COVID-19 (OR = 0.6148, 95% CI = 0.3955–0.9556, p = 0.0306). This suggests that the severity of MS may influence the patient’s immune status or physical condition and therefore their response to severe COVID-19 (**Figure 2**). In this process, rs10191329 was utilized as an instrumental variable.

In conclusion, we propose the hypothesis that rs10191329 acts as a mediator. It may indirectly affect the response of MS patients to severe COVID-19 by influencing the severity of MS and pathological characteristics. In this study, with MS as the exposure factor and COVID-19 as the outcome, a Mendelian randomization analysis was performed using a single SNP, rs10191329, as the instrumental variable. The results indicated an OR of 0.6148, which is less than 1, and a p-value of 0.0306. This finding suggests that individuals carrying the specific SNP rs10191329, compared to those who do not carry this SNP, have a lower risk of contracting severe COVID-19. Alternatively, if they do contract the virus, the severity of their illness is likely to be milder. Therefore, this suggests that MS patients with the rs10191329 allele may experience less severe disease manifestations if they contract COVID-19 (**Figure 2**). For SARS-CoV-2 infection and hospitalization with COVID-19, MR analysis did not find significant causality (OR = 1.0079, 95% CI = 0.8791–1.1555, p = 0.9104; OR = 0.8950, 95% CI = 0.6623–1.2094, p = 0.4701, respectively). This may imply that the severity and pathological characteristics of MS have less influence on these COVID-19 outcomes or that there are other confounding factors that were not considered. These studies allow for a better understanding of the role of genetic factors in the interaction between MS and COVID-19 and provide a basis for personalized treatment of MS patients.

The impact of rs10191329 on the severity and pathological characteristics of MS, as well as the potential influence of these biological changes on immune responses, warrants further investigation. Additionally, the hypothesis MS patients carrying the rs10191329 SNP may have a relatively reduced risk of severe COVID-19 infection requires rigorous examination. Furthermore, future studies should consider other factors that may influence the outcomes of COVID-19 in MS patients, including treatment, lifestyle, and environmental factors. These study samples are primarily of European ancestry, which may limit the wider relevance of our findings to other ethnic groups. Additionally, the small sample size may have reduced our ability to detect subtle genetic associations. Confounding factors, such as other genetic variants, environmental influences, and treatment differences, were not fully considered, which could affect the findings. Future research should include larger, multi-ethnic populations and control for these variables to improve accuracy.

We conducted a comprehensive analysis comparing individuals carrying the specific SNP rs10191329 with those who do not, considering MS as an exposure factor, to understand why these individuals have a lower risk of developing severe COVID-19, or, if they do contract the virus, they may experience milder disease severity. First, Root-Bernstein’s research indicates that an increase in neutrophils and the production of neutrophil extracellular traps are markers of heightened inflammation in severe COVID-19 and are associated with autoimmune complications (14). Second, the research of Nataf et al. reveals that the primary target of rs10191329 is the N-Acetylglucosamine Kinase (NAGK) gene, which plays a critical role in immune function by regulating the NOD2 pathway. NOD2 is a pattern recognition receptor that recognizes pathogen-associated molecular patterns in the innate immune response and initiates an immune response, thereby reducing the risk of viral infection (15). Thus, individuals carrying rs10191329 may, therefore, have a more effective immune response to SARS-CoV-2, helping control the virus and reduce the severity of the infection (15). Additionally, NAGK is involved in glucose metabolism, which is vital for immune cell function, especially during infections. Thus, MS patients carrying rs10191329 may have more effective immune cell function, thereby reducing the severity of COVID-19. Lastly, the research of Gasperi et al. indicates that rs10191329 is associated with brain atrophy in MS patients, potentially affecting their long-term clinical outcomes (16). Specifically, this suggests that the gene may play a role in neuroprotection. This means that in the face of COVID-19 infection, the central nervous system of these patients may be more resistant, reducing the overall impact of the virus on the body. Future research should focus on elucidating the precise mechanisms by which rs10191329 influences immune responses and neuroprotection in both MS and COVID-19. Clinical trials could explore targeted therapies that enhance NAGK function or modulate the NOD2 pathway, aiming to improve outcomes in MS patients with COVID-19. Moreover, longitudinal studies could assess whether rs10191329 carriers experience different long-term outcomes after COVID-19 infection, providing insights for personalized treatment strategies.

The study has shown that rs10191329 is associated with resilience in the CNS and neurodegenerative changes, which may suggest that this SNP influences the expression or function of related genes (17). However, researchers point out that while the discovery of rs10191329 is significant for understanding the pathophysiological processes associated with MS severity, it is unlikely that individual genotyping of this SNP will be used in clinical settings to guide disease management at this time (17). Another study found a statistically significant association between rs10191329, located within the DYSF-ZNF638 locus, and cross-sectional age-related MS severity (ARMSS) scores. Specifically, individuals carrying the A allele at this locus exhibited a 0.089-point higher ARMSS score, on average, 18.2 years after MS diagnosis (p = 3.6 × 10^−9^), suggesting that the A allele may be linked to increased MS severity by potentially influencing the expression of genes within this locus. Given that DYSF is involved in calcium-mediated membrane repair and regeneration, and ZNF638 in the transcriptional repression of retroviral DNA, any alterations in their expression could impact the function of the proteins they encode. This may, in turn, affect membrane maintenance in neural cells or transcriptional regulation processes relevant to MS pathology (18). Therefore, further experimental studies are required to elucidate how rs10191329 specifically affects gene expression and protein function and how these changes contribute to the progression of MS.

## Data Availability

The original contributions presented in the study are included in the article/**Supplementary Material**. Further inquiries can be directed to the corresponding authors.

## Supplementary material

Supplementary Figure 1 MR analysis estimating the impact of three COVID-19-related exposures on treatment outcomes in MS patients treated with IFNβ. The exposures considered include SARS-CoV-2 infection, hospitalized COVID-19, and severe COVID-19.

Supplementary Figure 2 MR analysis estimating the impact of treatment exposures in MS patients treated with IFNβ on three COVID-19-related outcomes.
