# Supplementary figures and images for: Multiple sclerosis and COVID-19: a bidirectional Mendelian randomization study
Source: Front Immunol. 2024 Oct 18;15:1451347. doi: 10.3389/fimmu.2024.1451347 (PMC11527686; doi:10.3389/fimmu.2024.1451347)

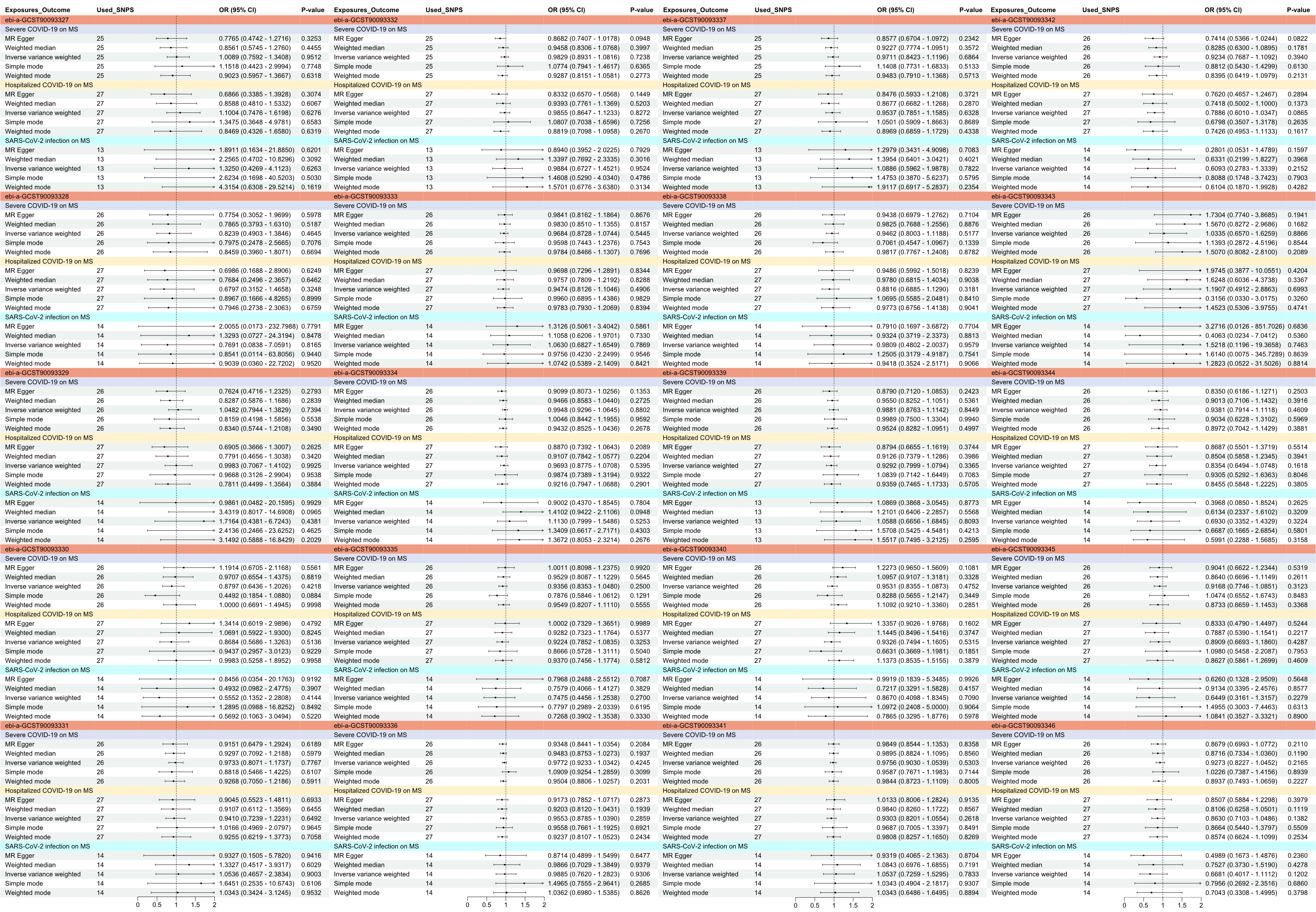

Supplement: Supplementary Figure 1 — MR analysis estimating the impact of three COVID-19-related exposures on treatment outcomes in MS patients treated with IFNβ. The exposures considered include SARS-CoV-2 infection, hospitalized COVID-19, and severe COVID-19. [file Image1.tif]

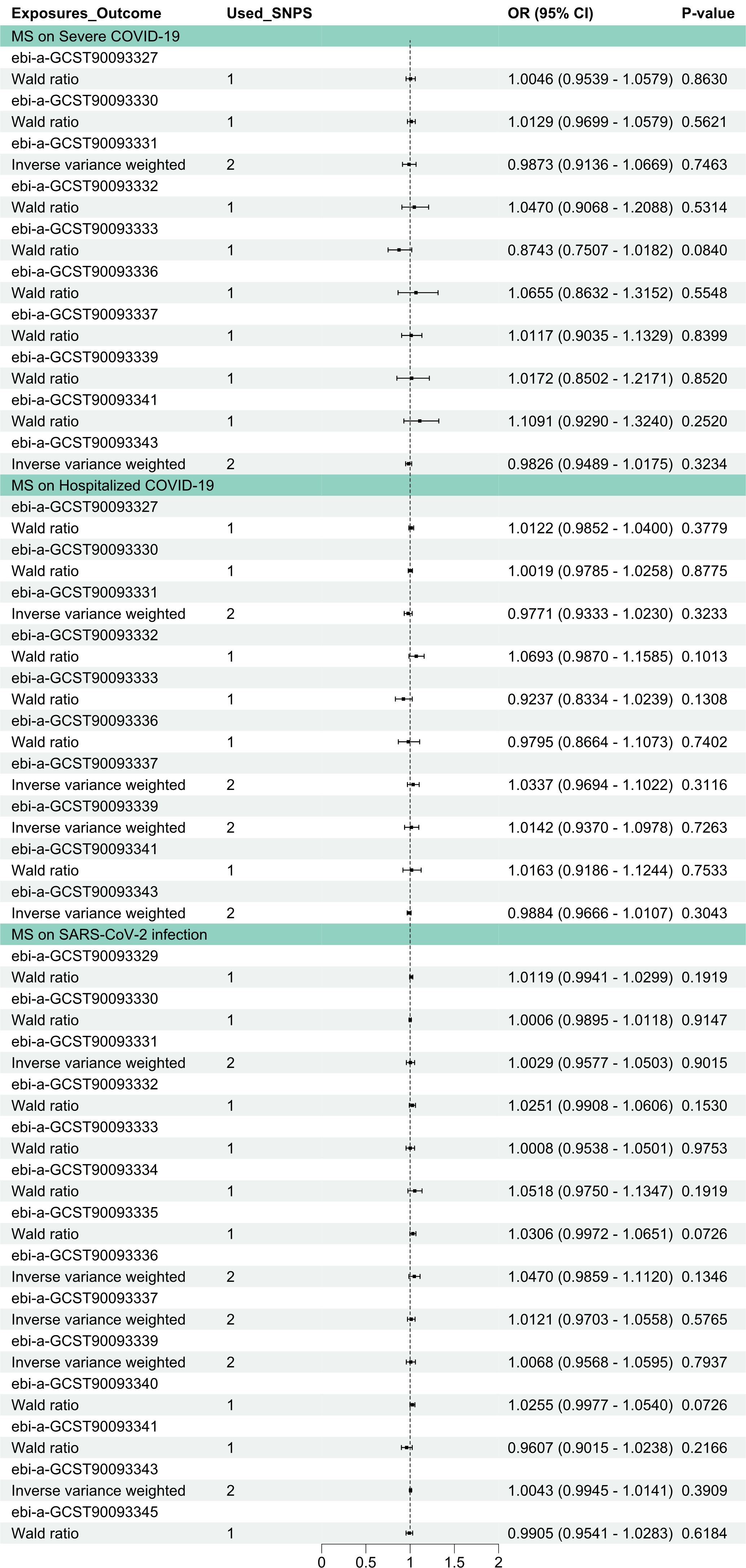

Supplement: Supplementary Figure 2 — MR analysis estimating the impact of treatment exposures in MS patients treated with IFNβ on three COVID-19-related outcomes. [file Image2.tif]
